# Hidden blood loss and bleeding characteristics in children with congenital scoliosis undergoing spinal osteotomies

**DOI:** 10.1007/s00264-024-06090-y

**Published:** 2024-01-17

**Authors:** Arimatias Raitio, Susanna Heiskanen, Venla Soini, Linda Helenius, Johanna Syvänen, Ilkka Helenius

**Affiliations:** 1 Department of Paediatric Surgery, Orthopaedics and Traumatology, University of Turku and Turku University Hospital, Turku, Finland; 2 Department of Surgery, Vaasa Central Hospital, Wellbeing Services County of Ostrobothnia, Vaasa, Finland; 3 Department of Anaesthesia and Intensive Care, University of Turku and Turku University Hospital, Turku, Finland; 4 Department of Orthopaedics and Traumatology, University of Helsinki and Helsinki University Hospital, Helsinki, Finland

**Keywords:** Scoliosis, Congenital abnormalities, Vertebrectomy, Blood loss, Osteotomy, Spine

## Abstract

**Purpose:**

Spinal osteotomies are often essential in the treatment of congenital scoliosis. Risk factors for bleeding in these patients needing extracavitatory approaches, especially hidden blood loss, are sparsely investigated. We aimed to investigate the bleeding characteristics and hidden blood loss in paediatric patients undergoing spinal osteotomies for congenital scoliosis.

**Methods:**

A retrospective analysis identified all patients with congenital scoliosis were retrospectively identified from the prospectively collected spine register from 2010 to 2022. Operative technique, perioperative laboratory results and imaging studies were extracted. The primary outcome was total blood loss including intraoperative, drain output and hidden blood loss.

**Results:**

Fifty-seven children (32 boys) with a mean age of 8.3 years underwent spinal osteotomy for congenital scoliosis. Posterolateral hemivertebrectomy was sufficient in 34 (59%) patients, while vertebral column resection (VCR) was required in 23 patients. Total bleeding averaged 792 (523) ml accounting for 42% of the estimated blood volume. Hidden blood loss accounted for 40% of total bleeding and 21% of estimated blood volume with a mean of 317 (256) ml. VCR was associated with greater intraoperative and total bleeding than hemivertebrectomies (*p* = 0.001 and 0.007, respectively). After adjusting for patient weight and fusion levels, hidden blood loss was larger in hemivertebrectomies (4.18 vs. 1.77 ml/kg/fused level, *p* = 0.049). In multivariable analysis, intraoperative blood loss was inversely correlated with preoperative erythrocyte levels. Younger age was associated with significantly greater drain, hidden and total blood loss.

**Conclusion:**

Hidden blood loss constitutes a significant portion (40%) of total bleeding in congenital scoliosis surgery. Younger age is a risk factor for bleeding and the hidden blood loss should be taken into consideration in their perioperative management.

## Introduction

Congenital scoliosis, which makes up to 10% of all scoliotic deformities, is defined as a spine deformity due to congenital vertebral malformation(s) [1]. A recent population-based study reported a total prevalence of 2.2 per 10,000 births [2], and pregestational diabetes appears to be a significant risk factor for these anomalies [3]. Congenital vertebral malformations are classified according to McMaster and Ohtsuka into formation defects (hemivertebra), segmentation defects (unilateral bar) and mixed deformities [4]. The spectrum varies from benign lesions requiring observation only to those causing severe congenital scoliosis or kyphosis with potentially life-threatening pulmonary insufficiency [5] or spinal cord deficits [6]. The most common indication for surgical intervention in congenital scoliosis is a fully segmented hemivertebra typically treated with hemivertebrectomy [7]. Segmentation defects, on the other hand, often necessitate an early anteroposterior spinal fusion, distraction-based spinal instrumentation, or thoracostomy and chest distraction [5, 8]. Vertebral column resection (VCR) is required for short, angular spinal deformities often due to underlying mixed type of congenital vertebral malformation [9].

Significant blood loss remains a major concern in spinal surgery [10], including surgery for congenital scoliosis [9]. Allogenic blood transfusions increase the risk of postoperative infection and delay recovery [11–13]. Total blood loss is composed of intraoperative bleeding, drain output [14, 15] and hidden blood loss (HBL). HBL is defined as blood accumulated intra- and postoperatively into the surrounding tissues and the surgical area [16, 17]. Risk factors for higher intraoperative bleeding include more severe spinal deformity, longer surgical time and more extensive fusion [13]. Several interventions have been proposed to mitigate intraoperative blood loss [11, 18–21], such as the application of gelatine matrix with human thrombin [14], the use of plasma blade [18] and the anterior approach only [11].

The hidden blood loss is thought to be caused by haemolysis, extravasation of blood into the tissues of the surgical site and subfascial haemorrhage [22, 23]. It was first introduced in knee arthroplasty by Sehat et al. in 2000 [24]. Since then, several studies have demonstrated that HBL constitutes a significant part of the total postoperative bleeding also in spinal fusion for scoliosis [16, 17, 22, 25]. Recently, Liu et al. reported that HBL accounted for over 40% of total blood loss in posterior hemivertebra resection for congenital scoliosis [26]. Hemivertebrectomy and posterior VCR are performed by an extracavitatory approach using costotransversectomy and an extra-pleural approach to enable a safe approach around the spine for osteotomy. Extracavitatory approach creates an additional surgical space where hidden blood loss might appear without clinical identification. As hemivertebrectomy is typically performed at an early age on patients with limited blood volumes, hidden blood loss may play an even more significant role in their perioperative management. There are no previous studies evaluating hidden blood loss in children undergoing all posterior vertebral column resection.

The aim of our study was to determine the intraoperative bleeding, drain output and hidden blood loss and risk factors for these bleeding characteristics in paediatric patients with congenital scoliosis undergoing hemivertebrectomy or VCR. We hypothesized that hemivertebrectomy necessitating a unilateral extracavitatory approach would mitigate the risk of hidden blood loss as compared to true VCR which mandates wider tissue exposure around the spine.

## Materials and methods

All patients undergoing spinal osteotomy for congenital scoliosis between years 2010 and 2022 were identified in the institutional prospectively collected paediatric spine register for this retrospective cohort study. Surgical procedures with growth-friendly implants such as growing rods were excluded. Indications for spinal osteotomies included fully segmented posterolateral hemivertebrae (hemivertebrectomy), congenital kyphosis and angular mixed-type congenital kyphoscoliosis (vertebral column resection) in the thoracolumbar spine (T1-S1). There were no exclusions for missing records as the data in the institutional spine register is collected prospectively.

Standardized preoperative clinical examination and testing included upright posteroanterior and lateral radiographs of the spine, CT images for evaluation of detailed bony anomalies and MRI of the spine and spinal cord prior to surgery. The pre and postoperative Cobb angles were measured by two paediatric orthopaedic surgeons (IH & JS). Routine laboratory testing included a standard coagulation profile (thrombocytes, activated partial thromboplastin time [aP-TT], international normalized ratio [INR], thrombin time [TT]), blood count including haemoglobin (Hb), electrolytes, creatinine and C-reactive protein (CRP).

### Anaesthetic management

The surgical and anaesthetic management were standardized. Patients were handled by consultant anaesthesiologists qualified in paediatric anaesthesia. The anaesthesia protocol was total intravenous anaesthesia, including propofol, remifentanil and dexmedetomidine infusions. Normothermia was maintained throughout the procedure. Mean arterial pressure was maintained at 65–75 mmHg throughout the operation and 24 h postoperatively, and noradrenaline infusion was utilized if necessary. An intravenous bolus of tranexamic acid (30 mg/kg, max 1500 mg) was administered before incision followed by an intraoperative infusion (10 mg/kg/h, max 500 mg/h). Allogenic red blood cell transfusion threshold was set at a haemoglobin level < 80 g/l. Fresh frozen plasma was given if the blood loss reached 50% of the estimated blood volume, and platelets were infused if blood loss exceeded 100% of the blood volume. The blood volume of the patient was estimated as 70 ml × weight (kg) (Table 1).

**Table 1:** Demographics and bleeding characteristics in congenital scoliosis. Values are given as mean (standard deviation) except for sex and drain usage, which are given in counts and also percentage for drain usage. *p* values are given for comparison between hemivertebrectomy and vertebral column resection groups

	All	Hemivertebrectomy (*n* = 34)	Vertebral column resection (*n* = 23)	*p* value
Age (y)	8.3 (5.7)	6.5 (5.3)	11.0 (5.4)	**0.005**
Sex (male / female)	32 / 25	20 / 14	12 / 11	0.078
Weight (kg)	33.0 (22.8)	25.1 (18.0)	45.8 (24.3)	**0.005**
Estimated blood volume (ml)	2370 (1593)	1789 (1264)	3328 (1645)	**0.002**
Fused vertebrae	6.5 (3.8)	6.2 (4.2)	7.1 (3.1)	0.099
Preoperative main curve (°)	39 (17)	41 (16)	37 (18)	0.283
Postoperative main curve (°)	12 (10)	12 (10)	14 (10)	0.530
Operative time (h)	3.8 (1.3)	3.5 (1.1)	4.4 (1.3)	**0.030**
Intraoperative blood loss (ml)	377 (378)	264 (294)	611 (432)	**0.001**
Drain output (ml)	313 (209)	339 (264)	299 (183)	0.812
Hidden blood loss (ml)	317 (256)	322 (244)	305 (286)	0.614
Total blood loss (ml)	792 (523)	648 (433)	1088 (581)	**0.007**
Cell saver (ml)	126 (81)	80 (86)	157 (64)	**0.045**
Drain usage (%)	25 (44)	9 (26)	16 (70)	**0.002**

Significant values are presented in bold

### Surgical protocol

**Fig. 1:**
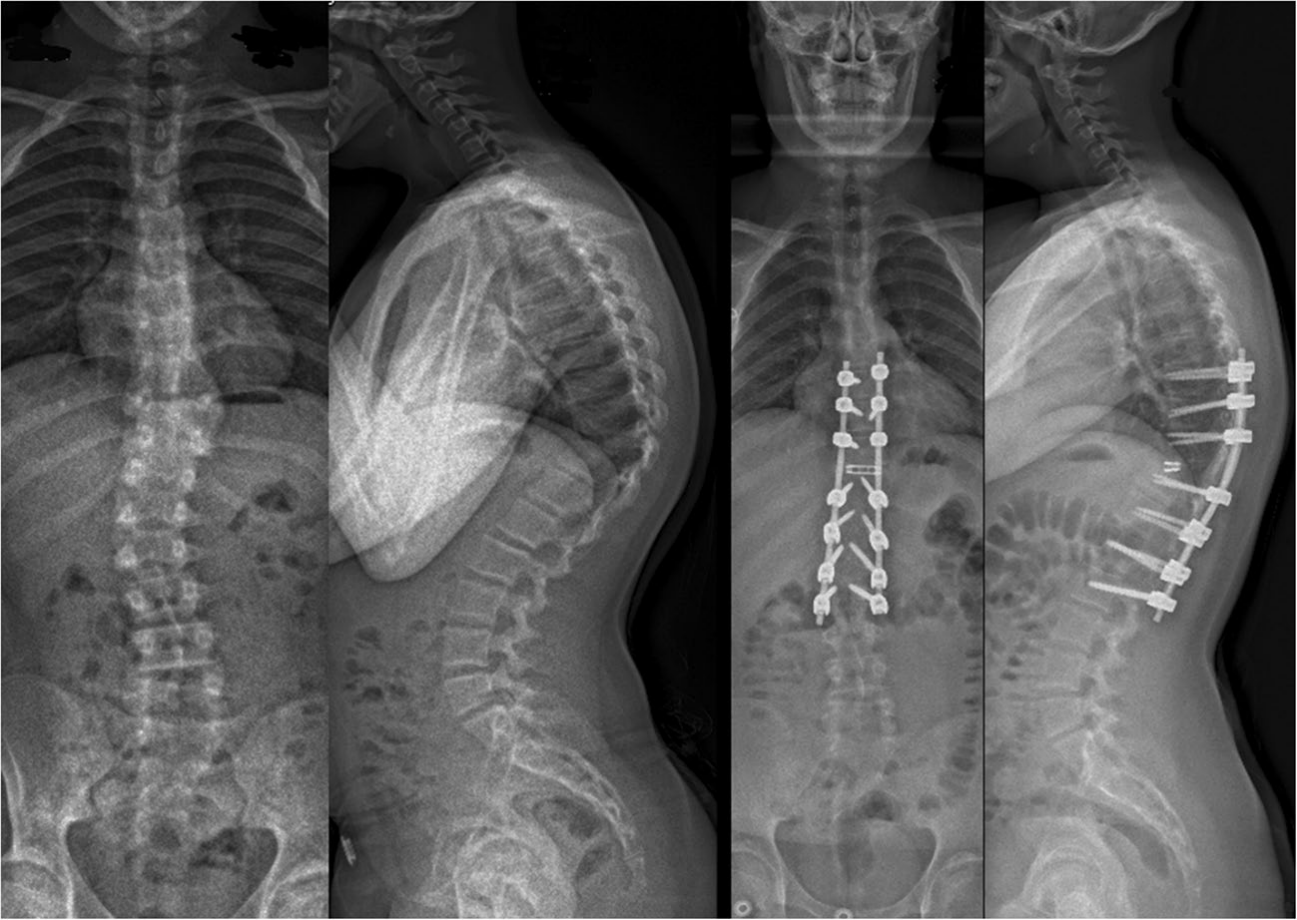
Calculation formula for hidden blood loss, adapted from Gross JB [27]

**Fig. 2:**
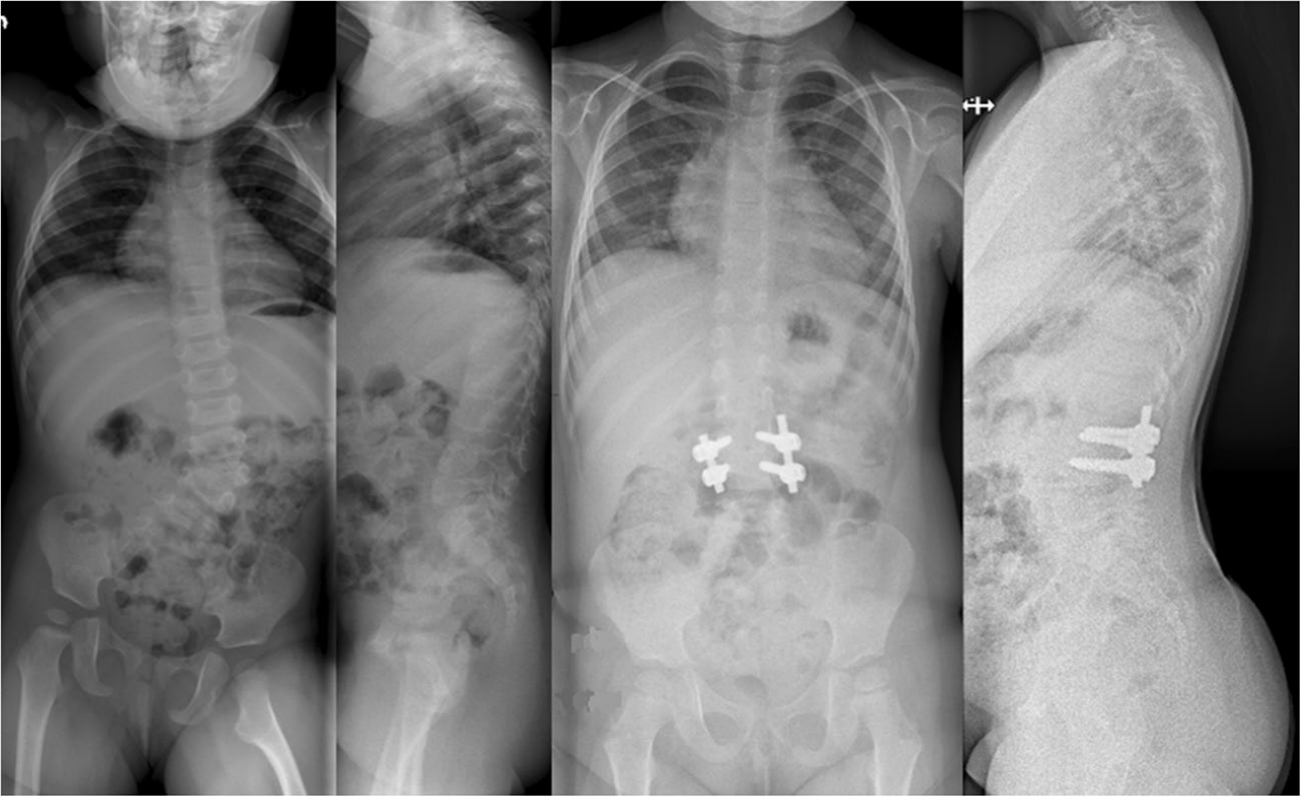
An 18-month-old girl with congenital scoliosis due to L3 posterolateral hemivertebra. L3 hemivertebrectomy with short-instrumented fusion from L2 to L4 with intraoperative blood loss of 170 ml. Standing posteroanterior and lateral preoperative and 2-year follow-up radiographs

Surgical procedure was planned based on the spine deformity and the number and anatomy of anomalous vertebrae on preoperative imaging: Posterolateral hemivertebrectomy using unilateral extracavitatory approach with monosegmental instrumentation was chosen for fully segmented hemivertebrae (hemivertebrectomy group, Fig. 1). Posterior vertebral column resection and posterior instrumentation as described by Lenke et al. [28], which requires a circumferential approach to the spine was performed in short angular and/or severe deformities (congenital kyphosis or mixed vertebral defects, vertebral column resection group, Fig. 2). The operations were performed by a single experienced paediatric orthopaedic spine surgeon (IH), together with another attending paediatric orthopaedic surgeon. The operative time was calculated from incision to wound closure. Posterior spinal exposure was performed with a diathermy blade to reduce blood loss. To address bleeding from bony pedicle screw channels, osteotomy sites and epidural bleeding not controlled by bipolar diathermy, a gelatin matrix with human thrombin (Floseal®, Baxter, US) was utilized, along with packing using oxidized regenerated cellulose (Surgicel Fibrillar®, Johnson & Johnson, US).

Routine neurophysiological monitoring (motor evoked potentials, somatosensory potentials, lumbar nerve root electromyography) was conducted every 20 min intraoperatively and at specific time points (incision, full exposure, after pedicle screw insertion, posterior elements removed, osteotomy completed, closure of the osteotomy and after wound closure). Antibiotic prophylaxis of cefuroxime and vancomycin was given at induction and continued for three doses postoperatively.

Intraoperative bleeding was estimated by measuring the amount of blood accumulated in the suction chamber from the surgical site and the amount of blood absorbed into the surgical drapes, with the subtraction of the fluid amount used for irrigation. The extent of intraoperative blood loss, 24-h postoperative drain output and the intra and postoperative blood transfusion rates were used as bleeding markers. Cell saver was utilized for intraoperative blood salvage in 19 (33%) patients. Subfascial Ch14 closed suction wound drain was routinely removed 24 h postoperatively.

### Outcomes

**Fig. 3:**
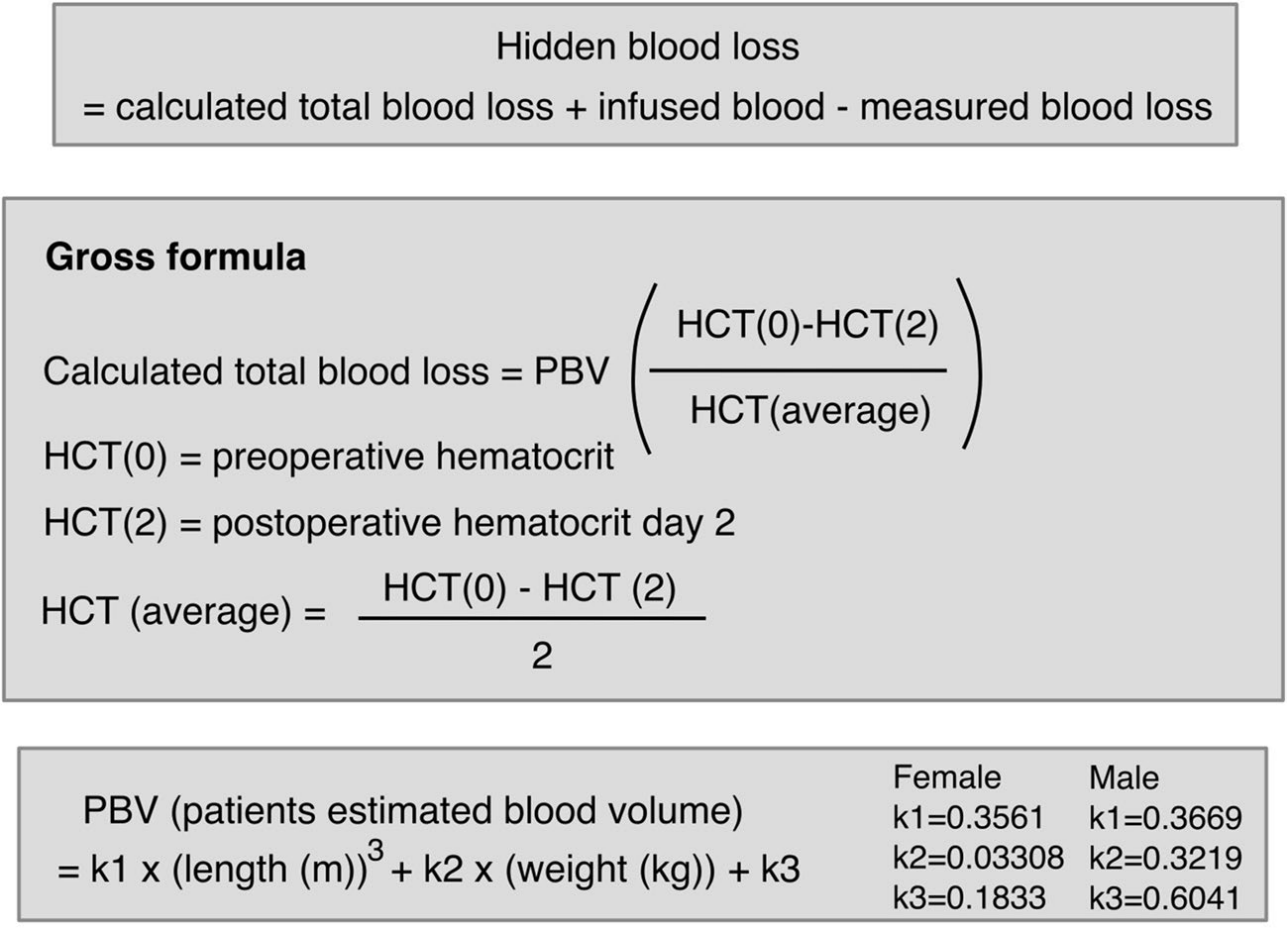
A 14-year-old boy with congenital kyphosis due to T10 posterior hemivertebra. Vertebral column resection of T10 via costotransversectomy approach, reconstruction with TLIF cage and instrumentation from T7 to L2 with intraoperative blood loss of 400 ml. Standing posteroanterior and lateral preoperative and 2-year follow-up radiographs

The primary outcome was total blood loss. Total measured blood loss included intraoperative blood loss and 24-h drain output. Total blood loss included measured blood loss and calculated HBL. Secondary outcomes included autologous (cell saver) and allogenic blood transfusion rate (red blood cell units). Hidden blood loss was calculated using a formula (Fig. 3) presented by Dr Gross [27].

### Statistical analysis

Normal assumption was tested from data distribution visually and with the Shapiro-Wilk test. For normally distributed continuous data linear fit and independent samples, a *t*-test was utilized. Categorical variables were tested with *χ*^2^-test. The Wilcoxon rank-sum test was used for nonparametric data. Variables in multivariable analysis were selected according to significance in univariate analyses. The Kruskal-Wallis test and pairwise comparisons were utilized in comparison of location (thoracic T1-T11 vs. thoracolumbar T12-L2 vs. lumbar L3-Sacrum) osteotomies. *p* < 0.05 was set as the limit for statistical significance in all analyses. Hidden blood loss was calculated according to principles demonstrated in previous publications [16, 22, 29, 30] and visualized in Fig. 3. The amount of bleeding was also divided by the patient weight (ml/kg) and further by the number of fused vertebrae (ml/kg/fused level) for additional analyses. Statistical analyses were performed with JMP Pro 16.2.

### Ethical considerations

The study was conducted in concordance with the Declaration of Helsinki and approved by the Ethical Committee at Turku University Hospital (ETMK 96/1801/2020).

## Results

**Fig. 4:**
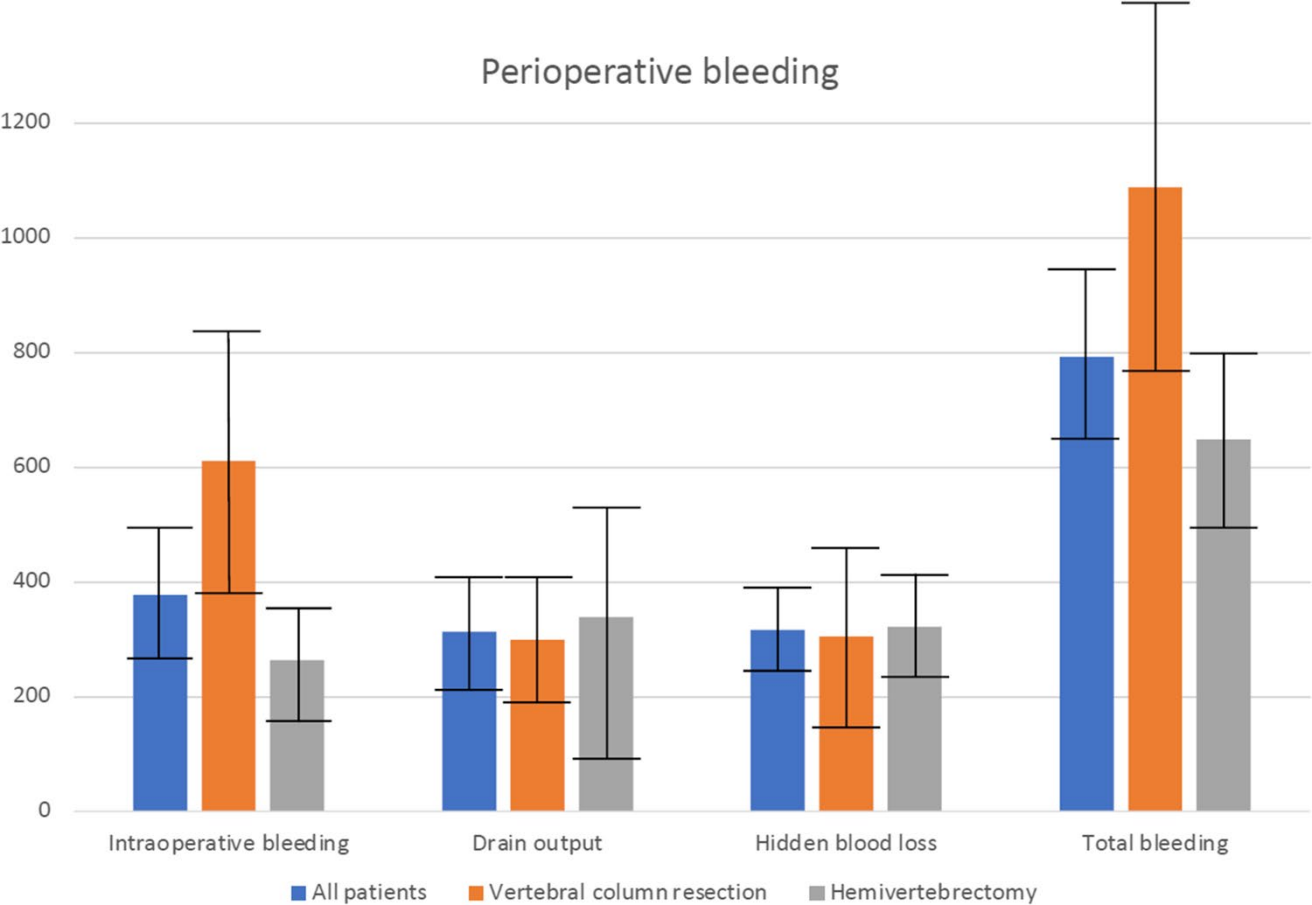
Visual comparison of bleeding subtypes, mean with 95% confidence intervals for bleeding in ml in all patients and VCR/ hemivertebrectomy subgroups

Fifty-seven consecutive patients (32 males, 25 females) with congenital scoliosis undergoing spinal osteotomy were identified in the register. The mean age was 8.3 years (standard deviation [SD]: 5.7, 95% confidence interval [CI]: [6.8–9.8]). Posterolateral hemivertebrectomy with a unilateral approach was sufficient in 34 patients (59%), while vertebral column resection with circumferential approach was required in 23 patients (31%). The number of fused levels was similar in VCR and hemivertebrectomy groups, 7.1 (SD 3.1, 95% CI [3.6–6.1]) and 6.2 (SD 4.2, 95% CI [3.1–4.0]) respectively (*p* = 0.10). Operative time was significantly longer in VCR (261 min [SD 80, 95% CI 216–305]) than in hemivertebrectomy group (213 min [SD 67, 95%CI 187–238], *p* = 0.03). However, there was no significant correlation with operative time and any type of bleeding. One patient experienced an intraoperative change in motor evoked potentials when a screw breakage occurred at the time of osteotomy closure resulting in medullary contusion. This neural deficit required 6 months for full motor recovery. Surgical treatment of congenital scoliosis was associated with significant bleeding, with a mean total blood loss of 792 ml (SD 523, 95% CI [641–942]) accounting for an average of 42% of estimated blood volume. Hidden blood loss accounted for 40% of total bleeding and 21% of estimated blood volume in this patient group with a mean of 317 ml (SD 256, 95% CI [242–391]) (Fig. 4).

Vertebral column resection, 611 ml (SD 432, 95% CI [381–841]) of intraoperative bleeding and 1088 ml (SD 581, 95% CI [778–1397] of total bleeding) resulted in significantly greater intraoperative and total blood loss than hemivertebrectomies, with means of 264 ml (SD 294, 95% CI [159–368]) for intraoperative and 648 ml (SD 433, 95% CI [495–802]) for total bleeding (*p* = 0.001 and *p* = 0.007, respectively, Table 1). Differences in intraoperative blood loss remained similar after adjusting for patient weight (15.4 ml/kg [SD 11, 95% CI [9.5–21.2]) vs. 10.7 ml/kg (SD 10.5, 95% CI [6.9–14.4], *p* = 0.033). After adjusting for weight and fusion levels, the hidden blood loss was significantly higher in patients undergoing hemivertebrectomy vs. VCR (4.18 ml/kg/fused level (SD 4.0, 95% CI [2.7–5.6] vs. 1.77 ml/kg/fused level (SD 2.2, 95% CI [0.62–2.92] ml/kg/fused level, *p* = 0.049). Patients undergoing vertebral column resection had larger amount of autologous blood transfused after cell saver salvage (157 ml [SD 64, 95% CI [108–206] vs. 80 ml (SD 86, 95% CI [−9–170]), *p* = 0.045). There were no bleeding-related complications in our cohort and no differences in allogenic blood transfusion rates with six (18%) patients in the hemivertebrectomy group and five (22%) in the vertebral column resection group receiving intraoperative allogenic blood transfusion and two in both groups postoperatively.

Patients with thoracic osteotomies (thoracic T1-T11) had significantly lower intraoperative (1.52 ml/kg/level [SD 1.10, 95% CI [1.09–1.96]), *p* = 0.0018) and total blood loss (4.51 ml/kg/level (SD 3.9, 95% CI [2.96–6.06]) ml/kg/level, *p* = 0.0028) as compared to patients with thoracolumbar (thoracolumbar junction T12-L2, 3.14 ml/kg/level (SD1.63, 95% CI [2.10–4.18]) and 8.37 ml/kg/level (SD 4.20, 95% CI [5.55–11.19]), respectively. The same phenomenon was observed in between thoracic and lumbar operations (L3-lumbosacral), mean for total bleeding 7.97 ml/kg/level (SD 5.49, 95% CI [4.04–11.9]), *p* = 0.0025. This difference was similarly significant in hemivertebrectomy subgroup, *p* = 0.043 for intraoperative and *p* = 0.0080 for total bleeding, yet not in patients undergoing VCR.

### Risk factors for bleeding

Patient age and number of fused vertebrae showed significant correlation with intraoperative and total blood loss in ml (correlation coefficient for intraoperative bleeding, 0.33 for age and 0.15 for fused vertebrae and for total bleeding, 0.45 for age and 0.31 for fused vertebrae). Patient gender, preoperative and postoperative major curve, or operative time did not affect the amount of bleeding measured in ml. In univariate risk factor analysis in ml/kg/level, patient age correlated inversely to all types of bleeding. Concerning preoperative laboratory levels, multiple correlations on bleeding were found as represented in Table 2. Preoperative haemoglobin, MCV, MCH and haematocrit levels showed negative correlations to bleeding. Multivariable regression analysis was performed with those factors with significant correlations in univariate analysis with bleeding outcomes in ml/kg/fused level. Intraoperative blood loss had an inverse correlation to preoperative erythrocyte level, *p* = 0.045. Younger age was significantly associated with greater drain blood loss, hidden blood loss and total blood loss. Multivariable regression analysis showed younger age at operation, male gender and lower INR levels to associate with more voluminous total bleeding in hemivertebrectomy patients (Table 3). In VCR patients, age at operation was a significant risk factor for drainage, hidden and total bleeding.

**Table 2:** Correlation coefficients for continuous variables in univariate analysis. Statistically significant factors were bolded and included in the multivariable analysis. When the correlation coefficient was less than 0.01, it was interpreted as 0

Risk factors ml/kg/level	IBL	DBL	HBL	TBL
Age at surgery	**−0.08 (*p*** **= 0.0432)**	**−0.56; (*p*** **< 0.001)**	**−0.43; (*p*** **< 0.001)**	**−0.47; (*p*** **< 0.001)**
Operative time (min)	0; (*p* = 0.9887)	0.05; (*p* = 0.4233)	−0.04; (*p* = 0.1794)	−0.01; (*p* = 0.440)
Preoperative main curve (°)	−0.01; (*p* = 0.5147)	−0; (*p* = 0.8217)	−0.02; (*p* = 0.2938	−0.04; (*p* = 0.1727)
Post. op main curve (°)	−0.04; (*p* = 0.1935)	−0.17; (*p* = 0.0857)	0; (*p* = 0.8111)	−0.017; (*p* = 0.3890)
Leukocytes	0.02; (*p* = 0.3506)	0.002; (*p* = 0.8703)	**0.17;** ***p*** **= 0.0039**	**0.14;** ***p*** **= 0.0093**
Erythrocytes	**−0.08; (*p*** **= 0.0446)**	−0.12; (*p* = 0.1548)	−0.01; (*p* = 0.6048	−0.06; (*p* = 0.1083)
Haemoglobin	−0.08; (*p* = 0.0536	**−0.36; (*p*** **= 0.0083)**	**−0.11; (*p*** **= 0.0205)**	**−0.18; (*p*** **= 0.0023)**
Haematocrit	**−0.09; (*p*** **= 0.0426)**	**−0.29; (*p*** **= 0.0201)**	−0.06; *p* = 0.0971	**−0.13; (*p*** **= 0.0110)**
MCV	−0.01; (*p* = 0.4668)	−0.13; (*p* = 0.1360)	**−0.13; (*p*** **= 0.0117)**	**−0.12; (*p*** **= 0.0166)**
MCH	−0.01; (*p* = 0.4747)	−0.**33;** (*p* = 0.0131)	**−0.23; (*p*** **< 0.001)**	**−0.19; (*p*** **= 0.002)**
Platelets	0.01; (*p* = 0.5620)	0.13; (*p* = 0.1350)	**0.13; (*p*** **= 0.0133)**	**0.11; (*p*** **= 0.0192)**
INR	−0.03; (*p* = 0.2328)	−0.02; (*p* = 0.5901)	**−0.17; (*p*** **= 0.0056)**	**−0.16; (*p*** **= 0.0057)**
AP-TT	0.02; (*p* = 0.5726)	−0.23; (*p* = 0.2297)	−0.13 (*p* = 0.1023)	−0.04; (*p* = 0.4061)
TT	0.01; (*p* = 0.5593)	−0.01; (*p* = 0.8020)	**0.27; (*p*** **= 0.0016)**	**0.20; (*p*** **= 0.0084)**
Krea	**−0.15; (*p*** **= 0.0134)**	−0.17; (*p* = 0.2389)	**0.30; (*p*** **< 0.001)**	**0.33; (*p*** **< 0.001)**

Significant values are presented in bold

Significant correlations are shown in bold

**Table 3:** Multivariable analysis for the risk factors for total blood loss in all patients and separately for hemivertebrectomy and vertebral column resection groups. Regression coefficients, *p*-values and *R*-square values of analyses

All patients (*n* = 57)	Age	Total blood loss
		−0.56; *p* < 0.001
	R-Square of fit	0.47
Hemivertebrectomy (*n* = 34)		
	Age	−0.47; *p* = 0.001
	Male sex	2.13; *p* = 0.002
	INR	−18.64; *p* = 0.037
	R-square of fit	0.63
Vertebral column resection (*n* = 23)		
	Age	−0.44; *p* < 0.001
	R-square of fit	0.56

## Discussion

Based on the current study, hidden blood loss is a significant source of bleeding in children undergoing spinal osteotomies for congenital scoliosis. Younger age at surgery portends a higher risk of perioperative bleeding. Hidden blood loss was significantly higher after adjusting for weight and fusion levels in patients undergoing hemivertebrectomy than in the vertebral column resection patients. The total blood loss including intraoperative, drain output and hidden blood loss represented 42% of our patients’ estimated blood volume. Therefore, it is crucial to take hidden blood loss into account, especially in younger patient groups needing hemivertebrectomy as their blood volume is more limited. Allogenic blood transfusion was required in 15 patients (26%). Early use of fresh frozen plasma could be one factor for reducing perioperative bleeding in children needing spinal osteotomies for congenital vertebral anomalies but requires further studies.

The literature regarding HBL in congenital scoliosis, surgery is limited. Recently, Liu et al. reported that HBL accounted for 41% of total blood loss in their series with 108 paediatric patients undergoing posterolateral hemivertebrectomy and posterior instrumentation [26]. Their mean total blood loss of 575 ml was also in line with the results of the current study as we observed a mean total blood loss of 648 ml in the hemivertebrectomy patients. However, they did not use a subfascial wound drain which may increase the estimated hidden blood loss. Liu et al. reported significant correlations between age, preoperative Cobb angle, operative time, number of fused levels and the total blood loss. Equivalent to their findings, the total blood loss in our cohort correlated with patient age and number of fused vertebrae. However, in our study, the total blood loss did not correlate with preoperative Cobb angle or operative time. After adjusting for patient weight, none of these variables was significantly associated with bleeding outcomes. Moreover, patient age was inversely correlated with weight-adjusted HBL and total blood loss. This study adds to the current literature that VCR for congenital spinal deformities in children is a significant risk factor for perioperative bleeding as compared to hemivertebrectomy alone.

We postulate that younger age is a risk factor for hidden blood loss as these patients have more elastic tissues with a capacity to expand before counter pressure will limit the amount of bleeding. Also, young patients present with 80% of the final adult spinal canal diameter from the age of two years [31]. Hence, bleeding from epidural veins might represent a potential source of blood loss during hemivertebrectomy or VCR. On the other hand, it must be noted that subfascial drains were only placed in 26% of the patients after posterolateral hemivertebrectomy (70% in VCR), and hemivertebrectomy patients were significantly younger. After adjusting for weight and fusion levels, the hidden blood loss was significantly higher in patients undergoing hemivertebrectomy vs. VCR. It is possible that this difference observed is at least partially explained by the different use of subfascial drain in these groups. Thus, postoperative bleeding cannot be measured in patients undergoing hemivertebrectomy but needs to be calculated using the hidden blood loss estimation.

Previously, the role of HBL has been studied in other types of paediatric spine surgery. Wang et al. reported that HBL constituted over 50% of total blood loss in posterior spinal fusion for adolescent idiopathic scoliosis [16]. Soini et al. compared bleeding characteristics in patients with neuromuscular and idiopathic scoliosis and observed that HBL accounts for 30% of total bleeding in both groups, but larger volumes were observed with neuromuscular scoliosis [25]. Based on the previous literature and the observations of the current study, it appears that HBL is a major source of bleeding in paediatric spine surgery, including spinal osteotomies for congenital scoliosis.

Thoracic hemivertebrectomies were associated with significantly lower intraoperative and total bleeding, which can be explained by a clear tissue plane (extrapleural approach) around the thoracic spine facilitating surgical exposure. In the thoracolumbar area, the diaphragm has to be dissected and in the lumbar spine, the psoas muscle may present a source of bleeding. To the best of our knowledge, this is the first report on this observation.

The role of red blood cells (erythrocytes) has been increasingly recognized in haemostasis, and the mechanisms continue to be elucidated. Erythrocytes are the main determinant of viscosity and flow dynamics. Also, red blood cells are an important and active part of the thrombotic process interacting with platelets in both a mechanical and biochemical fashion [32]. Therefore, it is logical that preoperative erythrocyte level was inversely correlated with intraoperative bleeding. However, further studies are needed to confirm the significance of this finding.

### Strengths and limitations

The strength of the current study is a relatively large number of consecutive children with congenital scoliosis undergoing spinal osteotomies with prospectively collected data. Also, perioperative examinations and surgical and anaesthetic management were standardized over the study period. This study is inherently limited by its retrospective design. However, the data in the spine register is prospectively collected resulting in complete dataset for all patients. One of the main limitations was the variability in subfascial wound drain use. All subfascial drains were removed routinely after 24 h. Up to 18% of drains will be contaminated, and the frequency of contaminated drain tips markedly increases after 24 h [33]. However, some bleeding may occur after this time point, and therefore hidden blood loss was calculated. Also, postoperative Hb was only collected for the first three postoperative days. The single-centre study design may limit the generalisability of these findings as surgical and anaesthetic management may vary between institutions.

## Conclusion

In conclusion, this study sheds light on the bleeding characteristics in children undergoing spinal osteotomies for congenital scoliosis. It reveals that hidden blood loss represents a substantial proportion, constituting 40% of the total blood loss in these patients, while total blood loss represented 42% of our children’s estimated blood volume. Furthermore, our findings highlight a significant inverse correlation between age and both hidden blood loss and total blood loss, underscoring the heightened risk of perioperative bleeding in younger patients. When adjusted for weight and fusion levels, hidden blood loss was larger in hemivertebrectomies than in vertebral column resections. This emphasizes the importance of recognizing and accounting for hidden blood loss, particularly in younger patients who possess limited blood volumes.

## Data Availability

The data that support the findings of this study are available on request from the corresponding author (IH).
